# Comparison of the efficacy and safety of flow diversion versus stent-assisted coiling in posterior circulation aneurysms: a systematic review and meta-analysis

**DOI:** 10.3389/fneur.2026.1813574

**Published:** 2026-04-23

**Authors:** Kuerban Maimaitiaili, Maimaitiaili Nuermaimaiti, Moming Abulaiti, Hongbo Pan, Ainipai Sidike, Yanbing Hu, Chao Li

**Affiliations:** 1 Department of Neurosurgery, The Second People’s Hospital of Kashgar Region, Kashgar, Xinjiang Uygur Autonomous Region, China; 2 Department of Neurology, The Second People’s Hospital of Kashgar Region, Kashgar, Xinjiang Uygur Autonomous Region, China; 3 Kashgar No. 8 Middle School, Kashgar, Xinjiang Uygur Autonomous Region, China; 4 Department of Neurosurgery, The People’s Hospital of Jiashi County, Kashgar, Xinjiang Uygur Autonomous Region, China

**Keywords:** FD, flow diversion, meta analysis, posterior circulation aneurysms, stent-assisted coiling (SAC)

## Abstract

**Introduction:**

Posterior circulation aneurysms are difficult to treat due to complex anatomy and perforator-rich territories, and the relative safety and efficacy of flow diversion (FD) versus stent-based strategies remain uncertain.

**Methods:**

This meta-analysis adhered to PRISMA and was registered in PROSPERO (No. 1302088). PubMed, Web of Science, EMBASE, and the Cochrane Library were searched from inception to September 24, 2025. We included patients with posterior circulation intracranial aneurysms treated with FD versus stents/SAC strategies. Dichotomous outcomes were pooled as risk ratios (RRs) with 95% CIs using Mantel–Haenszel methods.

**Results:**

Heterogeneity was evaluated with *I*^2^, applying fixed or random effects models at <50% or ≥50%, respectively. Eighteen retrospective cohort studies involving 1,486 patients were included (FD: 539; control: 947). FD and stents/SAC were comparable for favorable functional outcome (mRS 0–2) (RR = 1.04, 95% CI 0.91–1.19; *I*^2^ = 0.0%), follow-up complete occlusion (RR = 1.05, 95% CI 0.91–1.21; *I*^2^ = 0.0%), recanalization (RR = 1.61, 95% CI 0.39–6.65; *I*^2^ = 65.8%), complications (RR = 0.99, 95% CI 0.68–1.43; *I*^2^ = 0.0%), aneurysmal subarachnoid hemorrhage (aSAH) (RR = 0.55, 95% CI 0.25–1.22; *I*^2^ = 0.0%), and retreatment (RR = 0.71, 95% CI 0.33–1.49; *I*^2^ = 38.2%).

**Discussion:**

FD showed a lower rate of immediate complete occlusion (RR = 0.15, 95% CI 0.08–0.29; *I*^2^ = 15.2%) and a lower recurrence rate (RR = 0.13, 95% CI 0.04–0.43; *I*^2^ = 0.0%). Posterior circulation aneurysms treated with FD or stents/SAC show similar functional outcomes and overall safety. Stents/SAC achieved a higher immediate occlusion rate, whereas FD was associated with a lower recurrence rate.

## Introduction

Posterior circulation aneurysms are aneurysms arising from the vertebrobasilar system, including the vertebral arteries, basilar artery and their major branches such as the posterior inferior cerebellar artery (PICA), anterior inferior cerebellar artery (AICA), superior cerebellar artery (SCA), and posterior cerebral artery (PCA) (1). Compared with most anterior circulation lesions, posterior circulation aneurysms present greater clinical complexity because of the confined anatomical corridor, proximity to the brainstem, and the high density of perforating arteries. Rupture can result in subarachnoid hemorrhage with substantial morbidity and mortality, whereas unruptured aneurysms may present with ischemic events, brainstem compression, or progressive enlargement (2, 3). This challenge is particularly pronounced in dissecting or fusiform aneurysms of the vertebrobasilar system, where pathological changes often involve the parent vessel wall and may incorporate critical branches or perforators. Consequently, treatment requires a careful balance between effective lesion exclusion and preservation of posterior circulation perfusion to minimize ischemic complications. For patients in whom the parent artery must be preserved, reconstructive endovascular therapy has therefore become an important option (4, 5).

Current reconstructive endovascular strategies mainly include stent-based strategies and FD. SAC can achieve a relatively high rate of immediate post-procedural occlusion through coil packing, but it may still be associated with late recurrence or recanalization. In contrast, FD promotes progressive occlusion through flow remodeling and subsequent endothelialization and is theoretically more favorable for improving neck inflow and enhancing long-term stability (6). However, its use in the posterior circulation, especially in perforator-rich regions, still raises concerns about ischemic complications (7). Because existing evidence is largely derived from retrospective cohorts with substantial heterogeneity in lesion types and treatment indications, the comparative safety and effectiveness of FD versus stents/SAC for posterior circulation aneurysms remains controversial. Moreover, key angiographic and clinical endpoints have not been consistently defined across studies (7, 8). Against this background, we conducted a systematic review and meta-analysis to compare clinical and angiographic outcomes after FD versus stents/SAC in patients with posterior circulation aneurysms, with the aim of providing clearer evidence to inform individualized treatment strategies.

## Methods

We adhered to the PRISMA guideline for reporting this systematic review and meta-analysis (9). To enhance transparency, the protocol was registered with PROSPERO (registration No. 1302088).

### Search strategy

A systematic literature search was performed in PubMed, Web of Science, EMBASE, and the Cochrane Library, covering the period from database inception to September 24, 2025. The strategy incorporated both MeSH headings and free-text terms. The search strategy included (“Basilar artery” OR “Posterior cerebral artery” OR “Superior cerebellar artery” OR “Vertebral artery” OR “Posterior inferior cerebellar artery”) AND (“Intracranial Aneurysm”) AND (“Flow diverter”) AND (“Stent” OR “Stent-assisted coiling”). The full search strategy is provided in Supplementary File.

### Eligibility criteria

Inclusion criteria: Eligible participants were patients with posterior circulation intracranial aneurysms located in any of the following arteries: the basilar artery, vertebral artery (VA), PCA, SCA, PICA, or AICA. The intervention was treatment with FD, including the Pipeline Embolization Device and Pipeline Flex. The comparator was SAC or stent treatment. Studies were required to report at least one of the following outcomes: favorable functional outcome (mRS 0–2 at follow-up), immediate complete occlusion (complete aneurysm occlusion on post-procedural angiography), follow-up complete occlusion (complete aneurysm occlusion on the last imaging follow-up), recanalization (reopening or worsening of aneurysm occlusion during follow-up), recurrence (reappearance or regrowth of the treated aneurysm during follow-up), retreatment (any additional treatment for residual aneurysm, recurrence, or recanalization), aneurysmal subarachnoid hemorrhage (aSAH; subarachnoid hemorrhage caused by the target aneurysm), or complication (any treatment-related adverse event during the procedure or follow-up). Eligible study designs included prospective or retrospective observational studies and case series.

Exclusion criteria: Studies were excluded if they: (1) included aneurysms outside the posterior circulation; (2) failed to report outcomes of interest or provided insufficient/non-extractable data; or (3) were case reports, reviews, meta-analyses, editorials, or conference abstracts. For duplicate publications or overlapping cohorts, we included only the most recent study or the report with the largest sample size.

### Study selection and quality assessment

All references were imported into EndNote 21. Duplicate records were removed using the software’s automatic detection followed by manual verification. We first screened titles and abstracts to identify potentially eligible studies and then obtained full texts for eligibility assessment and final inclusion. Two investigators conducted study selection independently and cross-checked results afterward; any disagreement was resolved by consultation with a third investigator. The methodological quality of included observational studies was assessed using the Newcastle-Ottawa Scale (NOS) (10), which evaluates study quality across three domains (selection, comparability, and outcome). Total scores range from 0 to 9 points, with higher scores indicating better methodological quality.

### Data extraction

Two reviewers independently extracted data, with discrepancies adjudicated by a third reviewer. A standardized electronic extraction sheet was used to capture study descriptors (first author, publication year, and country) and participant characteristics, including age and study period.

### Statistical analysis

Statistical analyses were performed using Stata/MP 18.0. Treatment effects for dichotomous outcomes were summarized as RR with 95% confidence intervals (CIs). A *p*-value <0.05 was considered statistically significant. Pooled effect estimates were calculated using the Mantel–Haenszel method. Statistical heterogeneity among studies was assessed using the I^2^ statistic. A fixed-effect model was applied when heterogeneity was low (*I*^2^ < 50%), whereas a random-effects model was used when substantial heterogeneity was present (*I*^2^ ≥ 50%). Sensitivity analyses were conducted using a leave-one-out approach to evaluate the robustness of the pooled results and to determine whether any single study had an excessive influence on the overall effect estimate. Publication bias was evaluated using funnel plots and Egger’s regression test.

## Results

### Study selection

**Figure 1:**
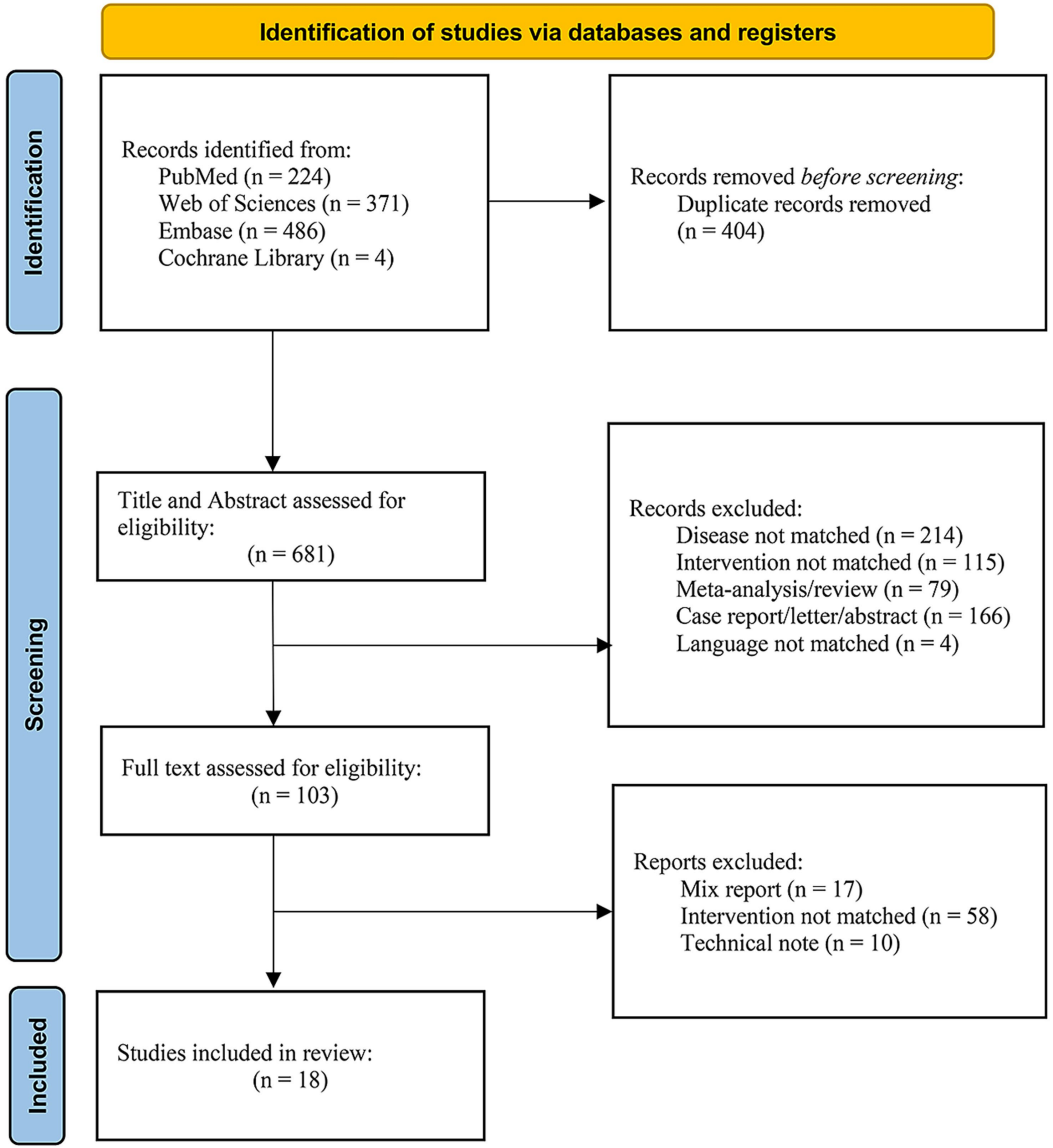
Flow diagram of literature search and study selection.

A total of 1,085 records were identified across the four databases. After removal of 404 duplicates by automated and manual screening, 681 records remained for title and abstract screening. Of these, 578 were excluded, leaving 103 articles for full-text review. Ultimately, 18 studies met all inclusion criteria (11–28). The study selection process is summarized in Figure 1.

### Characteristics of the included studies

A total of 18 retrospective cohort studies were included, comprising 1,486 patients, with 539 in the FD group and 947 in the control group. Studies were conducted primarily in China, Korea, and the United States. The case collection periods varied widely, spanning from 2005 to 2022. Most lesions were vertebral artery or vertebrobasilar system dissecting or fusiform aneurysms, with a small proportion of other posterior circulation aneurysms such as posterior cerebral artery aneurysms. With respect to interventions, the experimental group was treated mainly with FD, whereas the control group primarily underwent SAC or other conventional stent-based strategies. Baseline characteristics are summarized in Table 1.

**Table 1:** Characteristics of included studies and participants.

Author	Year	Country	Age	Study period	n1	Intervention	n2	Control	Disease
Joshua S. Catapano	2021	United States	53.0 ± 11.6	January 1999 to December 2019	29	FD	15	SAC	Vertebral artery dissecting aneurysms
D. Y. Cho	2019	Republic of Korea	51.37 ± 17.96	January 2009 to December 2017	7	FD	9	SAC	Unruptured intracranial vertebrobasilar dissecting aneurysm
Woo Cheul Cho	2023	Republic of Korea	52.6 ± 10.9	September 2008 to December 2020	13	FD	68	Stents/SAC	Vertebral artery dissecting aneurysms
Jiangli Han	2023	China	53.0 ± 8.89	January 2016 to December 2020	65	FD	59	SAC	Unruptured intracranial vertebral artery dissecting aneurysms
Zhe Ji	2024	China	58.97 ± 9.43	August 2013 to December 2021.	22	FD	25	Stents	Vertebrobasilar dolichoectasia aneurysms
Hyung Jun Kim	2024	Republic of Korea	54.2 ± 9.2	January 2012 to September 2022	25	FD	121	Stents	Unruptured vertebral artery dissecting aneurysms
Li Li	2022	China	52.0 ± 11.3	May 2014 to October 2019	25	FD	42	Stents/SAC	Intracranial vertebral artery unruptured dissecting aneurysms
Wenqiang Li	2022	China	50.8 ± 8.0	January 2014 to December 2018	22	FD	14	Stents	Unruptured vertebral artery fusiform aneurysms
Han San Oh	2023	Republic of Korea	56.63 ± 10.78	April 2009 to September 2021	24	FD	48	SAC	Unruptured vertebral artery dissecting aneurysms
Jiejun Wang	2019	China	49.15 ± 12.72	January 2014 to June 2018	42	PED	36	Stents	Non-saccular vertebrobasilar aneurysms
Qiaowei Wu	2022	China	55.43 ± 9.06	2014 to 2021	24	FD	42	SAC	Intradural large vertebrobasilar artery aneurysms
Qiaowei Wu	2023	China	57.03 ± 9.41	January 2014 to March 2022	36	FD	55	Stents	Intracranial vertebrobasilar trunk dissecting aneurysms
Tongfu Zhang	2024	China	55.56 ± 9.38	December 2011 to December 2022	51	FD	120	SAC	Unruptured intracranial vertebral artery dissection aneurysms
Yupeng Zhang	2019	China	52.36 ± 8.77	PED (11/2015–11/2016); SAC (3/2015–7/2017)	30	PED	64	SAC	Non-saccular, unruptured, intradural vertebral artery aneurysms
Yisen Zhang	2022	China	50.97 ± 13.91	January 2012 to December 2020	20	FD	16	Stents/SAC	Vertebrobasilar dissecting aneurysms
Yongxin Zhang	2016	China	NA	August 2010 to June 2014	56	FD	104	SAC	Large and giant aneurysms
Xinyu Li	2021	China	53.2 ± 10.6	February 2008 to October 2019	24	PED	18	SAC	Posterior cerebral artery
Peixi Liu	2023	China	53.3 ± 9.7	2005 to 2021	24	PED	91	SAC	Intracranial vertebral artery dissecting aneurysms

FD, Flow diversion; PED, Pipeline embolization device; SAC, Stent-assisted coiling.

### Meta-analysis results

#### Favorable functional outcome (mRS 0–2)

**Figure 2:**
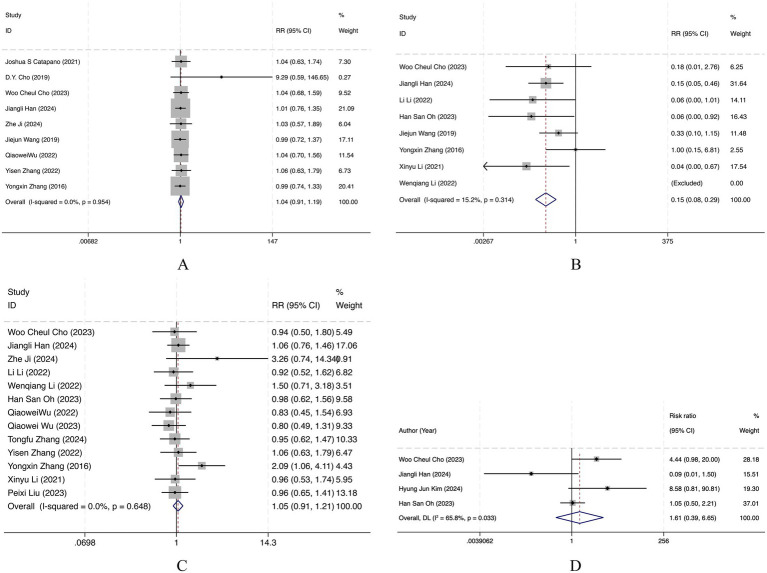
Forest plot of **(A)** favorable functional outcome (mRS 0–2); **(B)** immediate complete occlusion; **(C)** complete occlusion at follow-up; **(D)** recanalization.

Nine retrospective cohort studies were pooled for this outcome. There was no statistically significant difference between the FD group and the stents/SAC group in achieving a favorable functional outcome (mRS 0–2) (RR = 1.04, 95% CI 0.91–1.19). Between-study heterogeneity was negligible (*I*^2^ = 0.0%, *p* = 0.954), indicating consistent findings across studies (Figure 2A). Overall, FD and stents/SAC yielded comparable functional outcomes as measured by mRS 0–2.

#### Immediate complete occlusion

Seven retrospective cohort studies were included in this analysis, with one study excluded because the data were not applicable. The FD group had a significantly lower rate of immediate complete occlusion than the stents/SAC group (RR = 0.15, 95% CI 0.08–0.29). Heterogeneity was low (*I*^2^ = 15.2%, *p* = 0.314), suggesting generally consistent results (Figure 2B). Overall, FD was less likely than stents/SAC to achieve immediate complete occlusion.

#### Follow-up complete occlusion

Thirteen retrospective cohort studies were pooled for follow-up complete occlusion. No statistically significant difference was observed between FD and stents/SAC (RR = 1.05, 95% CI 0.91–1.21). Heterogeneity was negligible (*I*^2^ = 0.0%, *p* = 0.648), indicating consistent results across studies (Figure 2C). Overall, FD and stents/SAC achieved similar follow-up complete occlusion rates.

### Recanalization

Four retrospective cohort studies were included. There was no statistically significant difference between FD and stents/SAC in the rate of recanalization (RR = 1.61, 95% CI 0.39–6.65). Heterogeneity was substantial (*I*^2^ = 65.8%, *p* = 0.033), suggesting variability across studies (Figure 2D). Overall, no significant difference was observed between FD and stents/SAC with respect to recanalization.

### Recurrence

**Figure 3:**
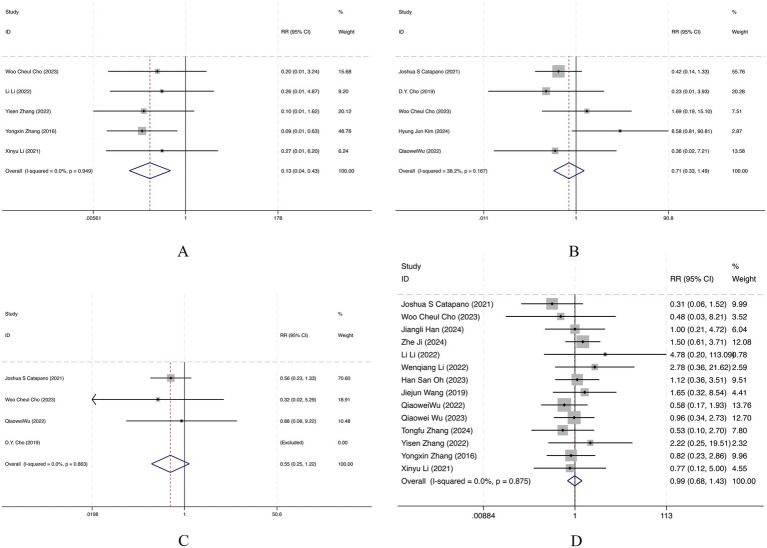
Forest plot of **(A)** recurrence; **(B)** retreatment; **(C)** aneurysmal subarachnoid hemorrhage (aSAH); **(D)** complications.

Five retrospective cohort studies were pooled for recurrence. The FD group had a significantly lower recurrence rate than the stents/SAC group (RR = 0.13, 95% CI 0.04–0.43). Heterogeneity was negligible (*I*^2^ = 0.0%, *p* = 0.949), indicating highly consistent findings (Figure 3A). Overall, FD was associated with a markedly reduced recurrence rate compared with stents/SAC.

### Retreatment

Five retrospective cohort studies were included. No statistically significant difference was found between FD and stents/SAC in retreatment (RR = 0.71, 95% CI 0.33–1.49). Heterogeneity was low to moderate (*I*^2^ = 38.2%, *p* = 0.167), suggesting generally consistent results (Figure 3B). Overall, retreatment rates did not differ significantly between FD and stents/SAC.

### Aneurysmal subarachnoid hemorrhage (aSAH)

Three retrospective cohort studies were included, with one study excluded because the data were not applicable. There was no statistically significant difference between FD and stents/SAC in the incidence of aSAH (RR = 0.55, 95% CI 0.25–1.22). Heterogeneity was negligible (*I*^2^ = 0.0%, *p* = 0.863), indicating consistent findings (Figure 3C). Overall, no significant difference was observed in the risk of aSAH between FD and stents/SAC.

### Complication

Fourteen retrospective cohort studies were pooled for complications. There was no statistically significant difference between FD and stents/SAC in complication rates (RR = 0.99, 95% CI 0.68–1.43). Heterogeneity was negligible (*I*^2^ = 0.0%, *p* = 0.875), indicating consistent findings (Figure 3D). Overall, complication risk did not differ significantly between FD and stents/SAC.

### Subgroup analysis by anatomical location

**Figure 4:**
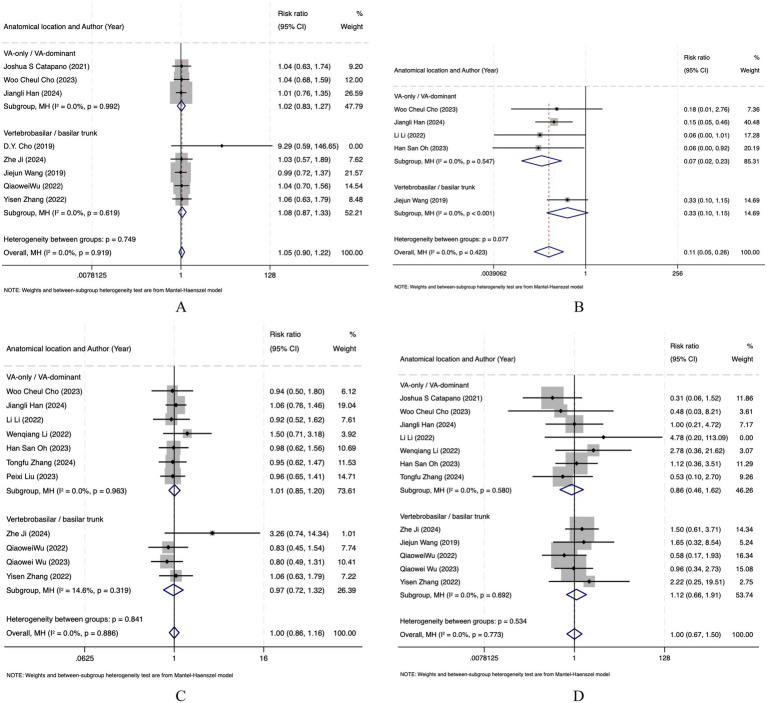
Forest plot of subgroup analysis for **(A)** favorable functional outcome (mRS 0–2); **(B)** immediate complete occlusion; **(C)** complete occlusion at follow-up; **(D)** complications.

Subgroup analyses were performed according to aneurysm location (VA-only/VA-dominant vs. vertebrobasilar/basilar trunk) when more than five studies were available for a given outcome. Because anatomical location was not clearly reported, Zhang et al. (26) was excluded from the analysis of favorable functional outcome (mRS 0–2), while both Zhang et al. (26) and Li et al. (27) were excluded from the analyses of immediate complete occlusion, follow-up complete occlusion, and complication. No significant subgroup difference was observed for favorable functional outcome (mRS 0–2), follow-up complete occlusion, or complication. For immediate complete occlusion, FD was associated with a significantly lower rate than stents/SAC in the VA-only/VA-dominant subgroup (RR = 0.07, 95% CI 0.02–0.23), whereas in the vertebrobasilar/basilar trunk subgroup, the result was based on a single study and did not reach statistical significance (RR = 0.33, 95% CI 0.10–1.15); no significant subgroup difference was found (*p* = 0.077). Overall, anatomical location did not appear to significantly influence the comparative effects of FD and stents/SAC (Figure 4).

### Sensitivity analysis

**Figure 5:**
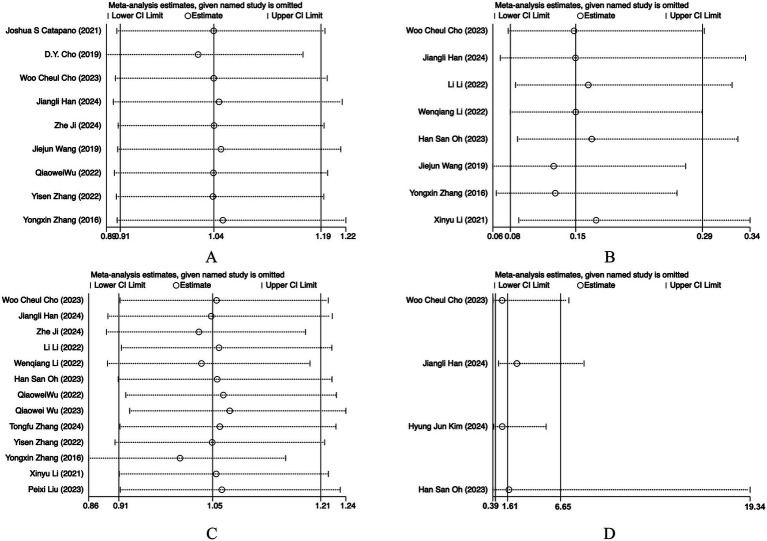
Sensitivity analyses of **(A)** favorable functional outcome (mRS 0–2); **(B)** immediate complete occlusion; **(C)** complete occlusion at follow-up; **(D)** recanalization.

**Figure 6:**
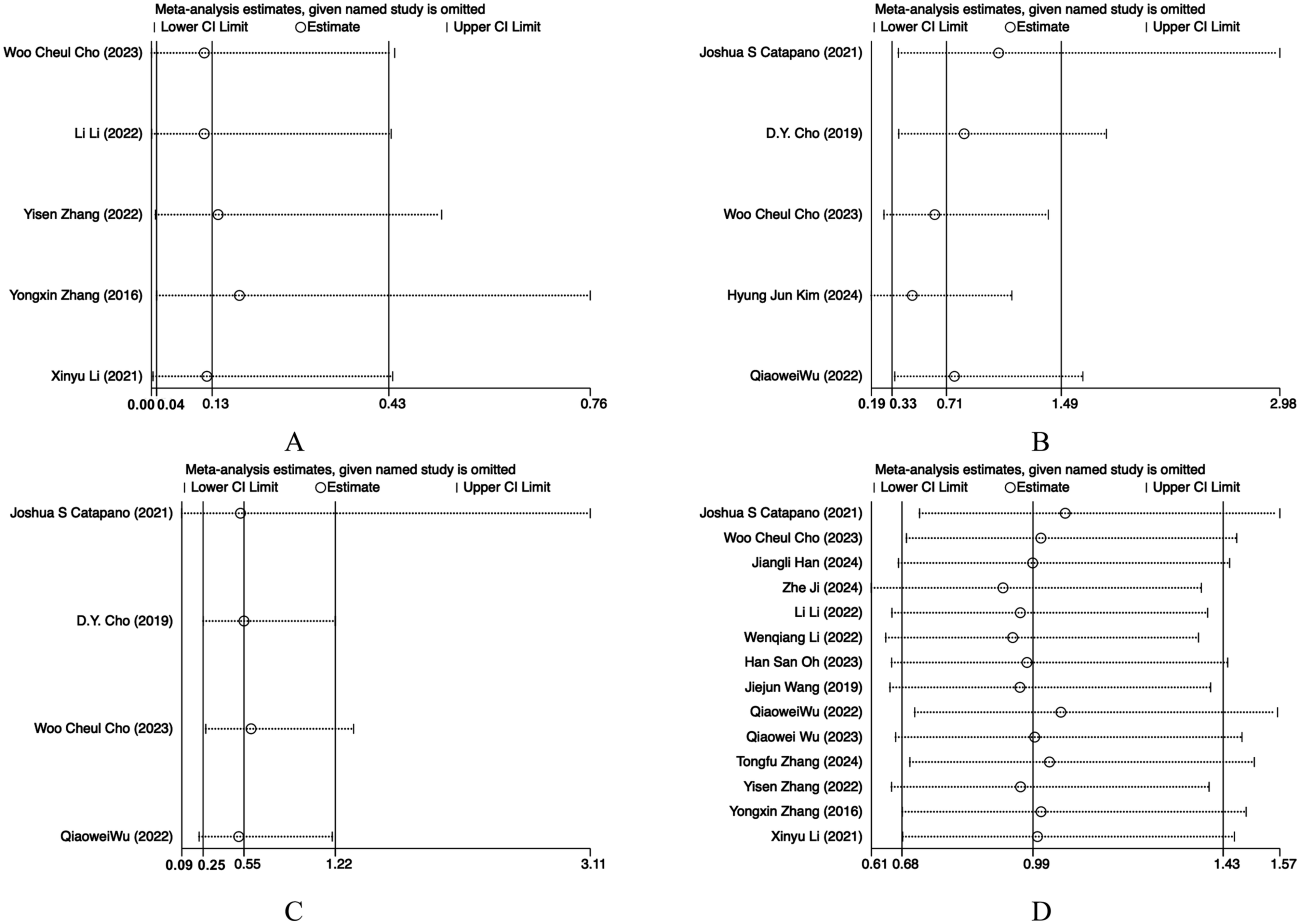
Sensitivity analyses of **(A)** recurrence; **(B)** retreatment; **(C)** aneurysmal subarachnoid hemorrhage (aSAH); **(D)** complications.

Sensitivity analyses were performed by sequentially omitting one study at a time. For all outcomes, the pooled estimates remained materially unchanged after exclusion of any single study, indicating that the results were generally robust. For recanalization, exclusion of the study by Han et al. (14) led to a reduction in heterogeneity from 65.8 to 59.5%, suggesting that this study may have contributed to the between-study variability; however, the overall effect estimate remained stable (Figures 5, 6).

### Quality assessment

The methodological quality of the included studies was assessed using the NOS. Overall, the included studies were of moderate to high quality, with NOS scores ranging from 5 to 9. Among the 18 studies, one scored 5 points, three scored 6 points, seven scored 7 points, five scored 8 points, and two scored 9 points. Most studies performed well in the selection and outcome domains, whereas comparability was relatively limited in several studies because of insufficient adjustment for potential confounding factors. In addition, the adequacy of follow-up was insufficient or unclear in some studies. The detailed NOS assessment results are presented in Table 2.

**Table 2:** Quality assessment of included studies using the Newcastle-Ottawa Scale.

Author	Year	Selection				Comparability	Outcome			Total
		Q1	Q2	Q3	Q4	Q5	Q6	Q7	Q8	
Joshua S. Catapano	2021	★	★	★	★	★	★	★		7
D. Y. Cho	2019	★	★	★	★		★	★		6
Woo Cheul Cho	2023	★	★	★	★	★	★	★	★	8
Jiangli Han	2023	★	★	★	★	★★	★	★	★	9
Zhe Ji	2024	★	★	★	★		★	★		6
Hyung Jun Kim	2024	★	★	★	★	★	★	★	★	8
Li Li	2022	★	★	★	★		★	★		6
Wenqiang Li	2022	★	★	★	★		★	★	★	7
Han San Oh	2023	★	★	★	★	★★	★	★	★	9
Jiejun Wang	2019	★	★	★	★		★	★	★	7
Qiaowei Wu	2022	★	★	★	★	★	★	★		7
Qiaowei Wu	2023	★	★	★	★	★	★	★		7
Tongfu Zhang	2024	★	★	★	★	★★	★	★		8
Yupeng Zhang	2019	★	★	★	★	★★	★	★		8
Yisen Zhang	2022	★	★	★	★		★	★	★	7
Yongxin Zhang	2016	★	★	★	★	★★	★	★		8
Xinyu Li	2021	★		★	★		★	★		5
Peixi Liu	2023	★		★	★	★	★	★	★	7

Q1: Representativeness of the exposed cohort; Q2: Selection of the non-exposed cohort; Q3: Ascertainment of exposure; Q4: Demonstration that outcome of interest was not present at start; Q5: Comparability of cohorts; Q6: Assessment of outcome; Q7: Was follow-up long enough for outcomes to occur; Q8: Adequacy of follow-up of cohorts.

### Publication bias

The funnel plot appeared largely symmetric, suggesting a low likelihood of substantial publication bias or small-study effects. Consistent with this visual assessment, Egger’s regression test was not significant (*p* = 0.511), providing no statistical evidence of publication bias.

## Discussion

This study systematically evaluated and compared clinical and angiographic outcomes in patients with posterior circulation aneurysms treated with FD versus stents/SAC. Overall, the pooled analyses suggested broadly similar performance across most key endpoints. No statistically significant differences were observed between FD and stents/SAC with respect to favorable functional outcome (mRS 0–2), follow-up complete occlusion, overall complication rate, the risk of aSAH, or retreatment, and heterogeneity across these outcomes was generally low, supporting good consistency of the findings. This overall pattern aligns with a recent review comparing FD and SAC, which likewise found that differences in functional outcomes and overall safety are often modest, with more pronounced contrasts typically emerging in angiographic endpoints (29). In contrast, our angiographic results showed a more distinctive profile. FD was associated with a significantly lower rate of immediate complete occlusion than stents/SAC but demonstrated a clear advantage in reducing recurrence. The recanalization outcome remained uncertain, reflected by substantial heterogeneity and wide confidence intervals. Prior reviews have similarly reported higher immediate occlusion rates with stents/SAC, whereas FD is characterized by delayed occlusion, with between-group differences often narrowing over follow-up and, in some studies, converging over time (30).

These findings are clinically plausible in light of the underlying treatment mechanisms and procedural pathways. Stents/SAC achieve immediate aneurysm sac filling and, with stent support, improve neck stability, which facilitates complete occlusion on post-procedural imaging. The therapeutic effect of FD depends primarily on flow remodeling that promotes intra-aneurysmal flow stagnation and progressive thrombosis, followed by neointimal growth and endothelialization across the aneurysm neck, resulting in parent vessel reconstruction. Accordingly, complete occlusion typically evolves over time rather than occurring immediately (31). Therefore, a lower immediate occlusion rate does not necessarily indicate inferior long-term efficacy. In our analysis, follow-up complete occlusion did not differ significantly between FD and stents/SAC, which is consistent with the delayed-occlusion profile of FD. The observed advantage of FD in recurrence may reflect more durable control of neck inflow and longer-term vessel reconstruction. A prior multicenter retrospective study also reported favorable long-term occlusion after FD for posterior circulation aneurysms and suggested that long-term stability may be superior to conventional reconstructive strategies (32). From a clinical decision-making perspective, these differences are relevant because they imply distinct therapeutic priorities. When rapid exclusion of the aneurysm from the circulation is required, such as in ruptured aneurysms or lesions considered at high risk of rupture, the higher immediate complete occlusion rate achieved with SAC may be advantageous (33). Conversely, when durable parent vessel reconstruction and long-term stability are the primary goals, FD may be more suitable, particularly for large, giant, fusiform, or dissecting aneurysms, consistent with its reconstructive concept (34). However, this angiographic advantage may not translate directly into a statistically significant reduction in retreatment, because retreatment is closely tied to clinical decision-making and is influenced by multiple factors beyond imaging findings, including follow-up intensity, thresholds for intervention, symptom evolution, and between-center practice variation. These differences can reduce comparability across studies and attenuate detectable between-group effects (29).

In addition, the substantial heterogeneity observed for recanalization may partly reflect inconsistent definitions of recanalization and recurrence, variation in follow-up duration, and nonuniform imaging assessment criteria across studies. Sensitivity analysis further suggested that the heterogeneity in recanalization may have been influenced, in part, by the study by Han et al. (14), as exclusion of this study led to a modest reduction in heterogeneity. Notably, this study was conducted in China, whereas the remaining studies were from Korea, suggesting that regional differences may have indirectly reflected variations in case selection, aneurysm characteristics, procedural techniques, or follow-up assessment. However, given the limited reduction in heterogeneity, the variability in recanalization is more likely multifactorial rather than driven by a single study. Importantly, although overall complication rates were similar in this meta-analysis, ischemia-related complications of posterior circulation FD, particularly perforator infarction, have been repeatedly highlighted in prior systematic reviews and meta-analyses. This underscores the need for careful patient selection, rigorous antiplatelet management, and close post-procedural surveillance when FD is applied in the posterior circulation (7). Furthermore, emerging endovascular strategies may help bridge the gap between early aneurysm protection and long-term durability. FD with adjunctive coiling has increasingly been explored as a hybrid approach intended to combine early occlusion with the long-term reconstructive benefits of flow diversion. A recent meta-analysis by Kesumayadi et al. (35) demonstrated that the addition of coils to pipeline embolization improved early occlusion rates and reduced retreatment, although this benefit was accompanied by increased procedure-related complications. These findings suggest that, in selected complex aneurysms, treatment strategies may evolve beyond a simple FD versus stents/SAC dichotomy toward a more individualized, lesion-specific approach.

This study has several limitations. First, all included studies were retrospective, which may introduce selection bias and residual confounding. Second, some cohorts were relatively small, limiting the precision of the pooled estimates. Third, although the subgroup analysis by anatomical location did not show significant interaction effects, incomplete reporting of lesion location in some studies limited the depth of stratified analysis. And differences in aneurysm types and locations within the posterior circulation, treatment strategies, follow-up duration, and imaging/occlusion criteria may have affected outcome assessment and contributed to heterogeneity. Therefore, large multicenter prospective studies with standardized imaging and long-term follow-up are needed to confirm the comparative efficacy and safety of flow diversion in posterior circulation aneurysms.

Overall, these results suggest that FD and stents/SAC are not simply interchangeable options with a uniform superiority of one over the other but rather represent strategies with different strengths depending on clinical priorities. When immediate complete occlusion is the primary objective, stents/SAC may be more advantageous. When the goal is to reduce recurrence risk and enhance long-term stability, FD may offer potential benefit. At the level of overall functional outcomes and safety, the two approaches appear broadly comparable. This conclusion is consistent with the general direction of current comparative evidence, but further validation in prospective studies with more standardized endpoint definitions remains necessary.

## Data Availability

The original contributions presented in the study are included in the article/Supplementary material, further inquiries can be directed to the corresponding authors.
