# Supplementary material for: Comparison of the efficacy and safety of flow diversion versus stent-assisted coiling in posterior circulation aneurysms: a systematic review and meta-analysis
Source: Front Neurol. 2026 Apr 23;17:1813574. doi: 10.3389/fneur.2026.1813574 (PMC13149105; doi:10.3389/fneur.2026.1813574)

**Supplementary Materials**

**Supplementary file 1:** Search strategy (PubMed)

| No. | Query | Results |
| --- | --- | --- |
| 1 | "Basilar Artery"[Mesh] | 7,468 |
| 2 | "Posterior Cerebral Artery"[Mesh] | 1,081 |
| 3 | "Vertebral Artery"[Mesh] | 10,402 |
| 4 | ((((((((((((((((((((((Basilar artery[Title/Abstract]) OR (Arteries, Basilar[Title/Abstract])) OR (Artery, Basilar[Title/Abstract])) OR (Basilar Arteries[Title/Abstract])) OR (posterior cerebral artery[Title/Abstract])) OR (PCA[Title/Abstract])) OR (Arteries, Posterior Cerebral[Title/Abstract])) OR (Artery, Posterior Cerebral[Title/Abstract])) OR (Cerebral Arteries, Posterior[Title/Abstract])) OR (Posterior Cerebral Arteries[Title/Abstract])) OR (Cerebral Artery, Posterior[Title/Abstract])) OR (superior cerebellar artery[Title/Abstract])) OR (SCA[Title/Abstract])) OR (vertebral artery[Title/Abstract])) OR (VA[Title/Abstract])) OR (Vertebrobasilar[Title/Abstract])) OR (Arteries, Vertebral[Title/Abstract])) OR (Artery, Vertebral[Title/Abstract])) OR (Vertebral Arteries[Title/Abstract])) OR (posterior inferior cerebellar artery[Title/Abstract])) OR (PICA[Title/Abstract])) OR (anterior inferior cerebellar artery[Title/Abstract])) OR (AICA[Title/Abstract]) | 156,570 |
| 5 | #1 OR #2 OR #3 OR #4 | 161,318 |
| 6 | "Intracranial Aneurysm"[Mesh] | 33,948 |
| 7 | ((((Intracranial Aneurysm[Title/Abstract]) OR (Aneurysm, Intracranial[Title/Abstract])) OR (Cerebral Aneurysm[Title/Abstract])) OR (Aneurysm, Cerebral[Title/Abstract])) OR (Aneurysm[Title/Abstract]) | 121,406 |
| 8 | #6 OR #7 | 131,803 |
| 9 | #5 AND #8 | 7,247 |
| 10 | (((((((((Flow diversion[Title/Abstract]) OR (flow diverter[Title/Abstract])) OR (Flow diverting stent[Title/Abstract])) OR (Pipeline Embolization Device[Title/Abstract])) OR (PED[Title/Abstract])) OR (FRED stent[Title/Abstract])) OR (p64 Flow Modulation Device[Title/Abstract])) OR (SILK flow diverter[Title/Abstract])) OR (Surpass flow diverter[Title/Abstract])) OR (Derivo Embolization Device[Title/Abstract]) | 8,104 |
| 11 | ((stent-assisted coiling[Title/Abstract]) OR (stent assisted coiling[Title/Abstract])) OR (stent[Title/Abstract]) | 100,303 |
| 12 | #9 AND #10 AND #11 | 224 |

**Supplementary file 2:** Funnel plot


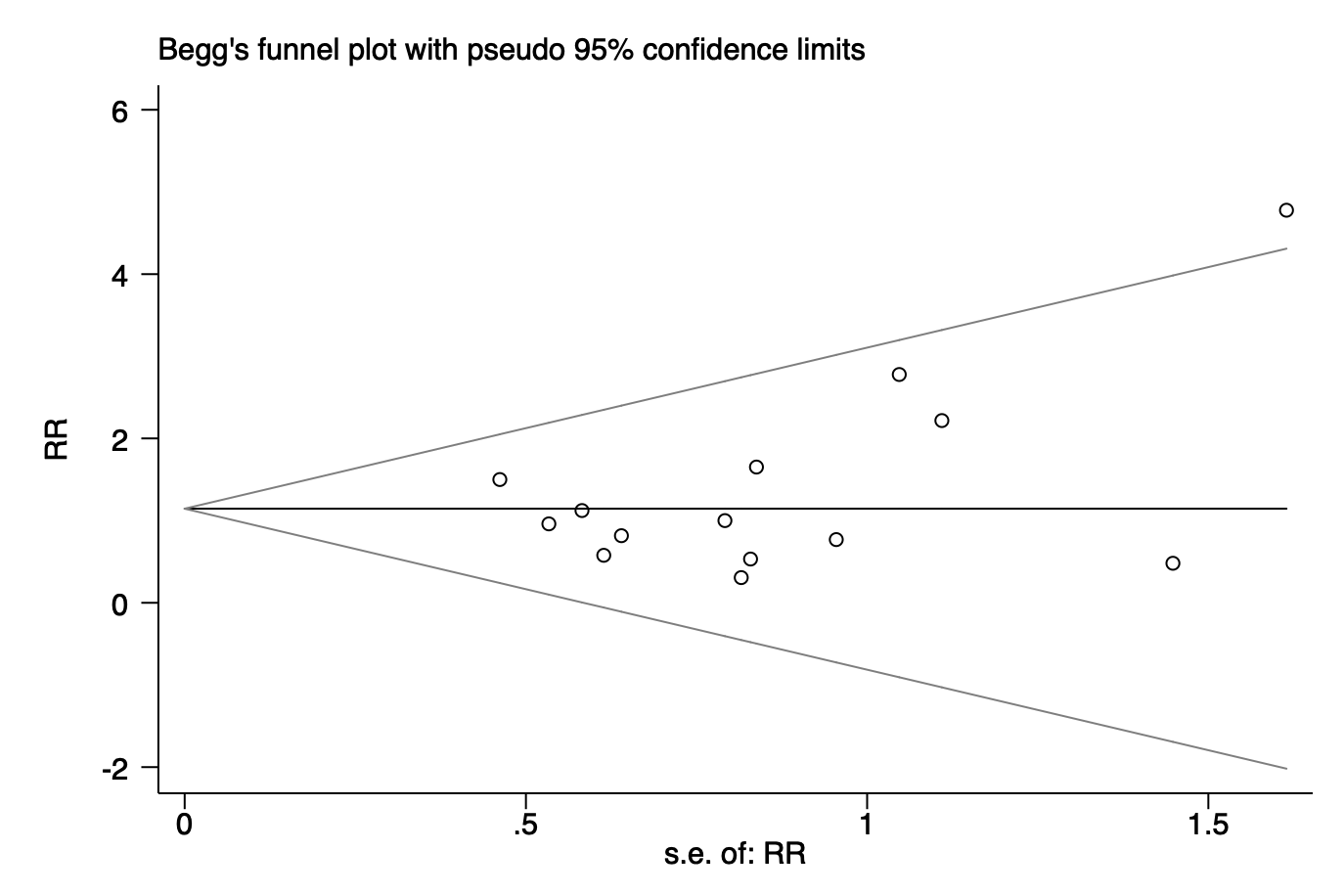

Supplement: Supplementary file 1 [file Table_1.DOCX]
